# Crucial roles of the Arp2/3 complex during mammalian corticogenesis

**DOI:** 10.1242/dev.130542

**Published:** 2016-08-01

**Authors:** Pei-Shan Wang, Fu-Sheng Chou, Sreekumar Ramachandran, Sheng Xia, Huei-Ying Chen, Fengli Guo, Praveen Suraneni, Brady J. Maher, Rong Li

**Affiliations:** 1 Stowers Institute for Medical Research, 1000 East 50th Street, Kansas City, MO 64110, USA; 2 Division of Newborn Medicine, Boston Children's Hospital, Harvard Medical School, 300 Longwood Ave., Boston, MA 02115, USA; 3 Department of Cell Biology, Johns Hopkins University School of Medicine, 855 North Wolfe Street, Baltimore, MD 21205, USA; 4 Lieber Institute for Brain Development, 855 North Wolfe Street, Baltimore, MD 21205, USA; 5 Division of Hematology/Oncology, Robert Lurie Comprehensive Cancer Center, Department of Medicine, Northwestern University, Chicago, IL 60611, USA; 6 Department of Psychiatry and Behavioral Sciences, Johns Hopkins School of Medicine, 4940 Eastern Ave., Baltimore, MD 21224, USA; 7 Department of Neuroscience, Johns Hopkins School of Medicine, 725 N. Wolfe St., Baltimore, MD 21205, USA; 8 Department of Chemical and Biomolecular Engineering, Johns Hopkins University, 3400 N Charles St., Baltimore, MD 21218, USA

**Keywords:** Arp2/3 complex, Actin, Cortical development, Neurogenesis, Neuronal migration, Radial glia, Mouse

## Abstract

The polarity and organization of radial glial cells (RGCs), which serve as both stem cells and scaffolds for neuronal migration, are crucial for cortical development. However, the cytoskeletal mechanisms that drive radial glial outgrowth and maintain RGC polarity remain poorly understood. Here, we show that the Arp2/3 complex – the unique actin nucleator that produces branched actin networks – plays essential roles in RGC polarity and morphogenesis. Disruption of the Arp2/3 complex in murine RGCs retards process outgrowth toward the basal surface and impairs apical polarity and adherens junctions. Whereas the former is correlated with an abnormal actin-based leading edge, the latter is consistent with blockage in membrane trafficking. These defects result in altered cell fate, disrupted cortical lamination and abnormal angiogenesis. In addition, we present evidence that the Arp2/3 complex is a cell-autonomous regulator of neuronal migration. Our data suggest that Arp2/3-mediated actin assembly might be particularly important for neuronal cell motility in a soft or poorly adhesive matrix environment.

## Abstract (precis)

**Summary:** During mouse cortical development, the Arp2/3 actin branching complex regulates process formation and the maintenance of radial glial cell polarity, as well as affecting neuronal migration.

## INTRODUCTION

During embryonic neurogenesis, radial glia cells (RGCs), a population of highly polarized stem cells, give rise to most cortical neurons and also serve as their radial migration scaffold (reviewed by Ayala et al., 2007; Rakic, 2003b). The apically located RGCs maintain their marked apicobasal polarity with a short apical process that forms an end-foot attached to the ventricular zone (VZ) and a long basal (radial) process that contacts the pia surface, where it is anchored to the basement membrane (Schmechel and Rakic, 1979). The division of RGCs can be either symmetrical, producing two daughter RGCs (to expand the radial glial population), or asymmetrical, producing an RGC and either a daughter neuron (postmitotic) or an intermediate progenitor cell (IPC; mitotic) (Anthony et al., 2004; Malatesta et al., 2000; Miyata et al., 2001; Noctor et al., 2004, 2008). The neuron then migrates along the RGC basal process to the cortical plate (CP) and completes the differentiation process. The IPC may undergo multiple rounds of symmetric division to expand in number or to generate neurons (LaMonica et al., 2013). The apical end-feet of RGCs are anchored to each other through adherens junctions (AJs), and this is essential to maintain VZ integrity and RGC identity (Buchman and Tsai, 2007). The basal processes are highly dynamic and are thought to be involved in neuronal positioning (Ayala et al., 2007; Yokota et al., 2009). Abnormalities in RGC polarity would therefore affect both neurogenesis and migration, and may underlie neurodevelopmental disorders and brain tumor (Götz and Huttner, 2005; Rakic, 2003a; Taylor et al., 2005).

Despite its importance in brain development, the molecular mechanisms that regulate RGC polarity, adhesion and basal extension are poorly understood. Recent studies indicate that adenomatous polyposis coli (Apc) and Cdc42 localize at the tip of RGC basal processes and are required for RGCs to respond to polarity maintenance cues, such as neuregulin 1, and to regulate basal end-feet attachment to the pia surface, respectively (Yokota et al., 2010, 2009). On the ventricular side, the AJ components (cadherins and catenins) and proteins with conserved roles in the regulation of cell polarity and asymmetric cell division (e.g. Par3, Par6, aPKC, Cdc42, Numb) localize to the apical membrane and play crucial roles in RGC adhesion and polarity. Loss of these apical polarity proteins results in the mislocalization or loss of AJs, formation of neuroblastic rosettes, abnormal mitotic entry location, abnormal IPC fate, premature neuronal differentiation and depletion of neural progenitors (Buchman and Tsai, 2007; Bultje et al., 2009; Cappello et al., 2006; Rašin et al., 2007; Zhang et al., 2010).

A likely effector of the above polarity proteins is the actin cytoskeleton, which is also known to play key roles in cell-cell and cell-matrix interactions. A key step in actin polymerization, and thus an important point of regulation *in vivo*, is the nucleation of filaments. The evolutionarily conserved Arp2/3 complex nucleates branched actin networks. The Arp2/3 complex consists of a stable and stoichiometric assembly of seven polypeptides, including Arp2 (Actr2), Arp3 (Actr3) and Arpc1-5. In the nervous system, the Arp2/3 complex has been shown to be involved in growth cone motility and axon guidance, development of dendritic spines and synapses, and memory decay (Hadziselimovic et al., 2014; Kim et al., 2013; Koch et al., 2014; Korobova and Svitkina, 2008; Lippi et al., 2011; Nakamura et al., 2011; Norris et al., 2009; Rocca et al., 2013; San Miguel-Ruiz and Letourneau, 2014; Spillane et al., 2011; Strasser et al., 2004; Wegner et al., 2008; Yang et al., 2012). The expression level of Arp2/3 complex subunits has been linked to human neurodevelopmental disorders, such as Down syndrome (Weitzdoerfer et al., 2002) and brain tumor (Liu et al., 2013). In the former study, a significant reduction of the 20 kDa subunit of the Arp2/3 complex was observed in fetal Down syndrome brain; in the latter, a positive correlation between the expression of Arp2/3 subunits and the malignancy of glioma specimens was described. Although the Arp2/3 complex has been studied in various types of cultured cells, its *in vivo* function in mammalian neurogenesis has not been elucidated owing to the early embryonic lethality that results from its disruption in mice (Suraneni et al., 2012; Yae et al., 2006). Cdc42 and RhoA, upstream regulators of the Arp2/3 complex, have been shown to control RGC basal process extension and to regulate RGC apical adhesion and cell fate (Cappello et al., 2006, 2012; Yokota et al., 2010), raising the possibility that the Arp2/3 complex might be crucial for brain development by regulating RGC polarity and morphogenesis.

In this study, we took a conditional gene ablation approach to dissect the function of the Arp2/3 complex during mouse embryonic cortical development. We show that mouse embryos in which *Arpc2* is disrupted exhibit abnormal corticogenesis. This phenotype is due to defects in RGC apicobasal polarity and radial glial extension, leading to impaired angiogenesis, neurogenesis and neuronal migration. In addition, we show that the Arp2/3 complex is a cell-autonomous regulatory factor for neuronal migration. We also demonstrate that the Arp2/3 complex plays a role in cellular responsiveness to biochemical and mechanical properties of the environment.

## RESULTS

### Conditional ablation of *Arpc2* disrupts cortical development

Previous studies demonstrated that conventional gene disruption of the Arpc3 subunit of the Arp2/3 complex results in early embryonic lethality (Suraneni et al., 2012; Yae et al., 2006). We therefore developed a conditional Arp2/3 complex-deficient mouse that allows the function of the complex to be studied at specific developmental stages or in specific tissues. This mouse, purchased originally as a flipper gene-trap line from the Sanger Institute (UK), has a floxed allele of *Arpc2* whereby Cre-mediated recombination truncates the expression of the protein at amino acid 182 (Fig. S1A). Arpc2 is one of the two central scaffolding subunits of the Arp2/3 complex. Biochemical studies of the Arp2/3 complex in both human and yeast have shown that ARPC2 is essential for the integrity of the entire complex (Goley et al., 2010; Winter et al., 1999). The truncation removes the helix-helix interaction required for the ARPC2/ARPC4 central scaffolds of the complex and mother filament interaction (Daugherty and Goode, 2008; Gournier et al., 2001; Robinson et al., 2001) and is thus predicted to result in complex-complex disruption. To confirm that this truncation results in a null allele, we created the analogous mutation in budding yeast ARPC2 (Arc35) and confirmed that it produces an Arp2/3 complex null phenotype (Fig. S1B). Subsequent analysis of the *Arpc2^f/f^ Nestin-Cre* mutant mouse brains confirmed the lack of Arpc2 protein expression and of localization of the Arp2/3 complex (see below).

To elucidate the function of the Arp2/3 complex in cortical development, we disrupted Arpc2 by crossing *Arpc2^f/f^* with a *Nestin-Cre* line (Cre recombinase driven by the nestin enhancer and the human β-globin basal promoter together with the 0.3 kb intron 2) in order to express Cre in the developing RGCs. The *Nestin-Cre* transgene induced widespread recombination in the CNS neural progenitors from around embryonic day (E) 12.5, and loss of Arpc2 was evident in the cortices of *Arpc2^f/f^ Nestin-Cre* embryos after 13.5 days of gestation (Fig. S2A, Fig. S4A). We observed severe intraventricular hemorrhage (IVH) in *Arpc2^f/f^ Nestin-Cre* mouse embryos at E15.5 (Fig. S2B). In addition, thinning of the lateral cortices and enlargement of the lateral ventricles were also apparent from E14.5 (Fig. S2C,D).

To further verify the roles of the Arp2/3 complex in cortical development, we also disrupted *Arpc2* by crossing with an *Emx1-Cre* line, as *Emx1* expression is more restricted to dorsal cortical neural progenitors (De Pietri Tonelli et al., 2008). IVH was again observed in the *Arpc2^f/f^ Emx1-Cre* mouse embryos at E14.5 (Fig. S2E). Interestingly, thinning of the lateral cortex and enlargement of the lateral ventricles were not as obvious at E14.5 in the *Arpc2^f/f^ Emx1-Cre* as compared with the *Arpc2^f/f^ Nestin-Cre* embryonic brain (Fig. S2E). This suggests that the thinning of the lateral cortices and the enlargement of the lateral ventricles in *Arpc2^f/f^ Nestin-Cre* mouse embryos might be due to pressure generated from severe hydrocephalus.

### Accelerated differentiation of Arpc2-depleted RGCs in association with decreased proliferation and increased apoptosis

**Fig. 1.**
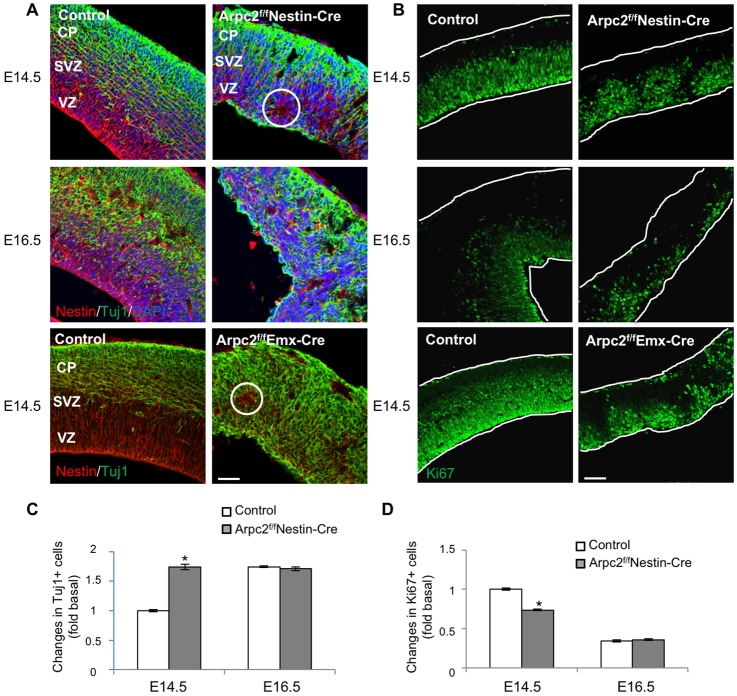
**Premature neuronal differentiation and depletion of neural progenitors following *Arpc2* deletion.** (A) RGCs and cells of the neuronal lineage in E14.5 and E16.5 cortices were labeled with anti-nestin (neural stem cell-specific) and anti-Tuj1 (neuronal lineage-specific) antibodies. In control cortices, a Tuj1-negative ventricular zone was clearly evident. By contrast, Arpc2-deficient cortices lack the Tuj1-negative ventricular zone. At E14.5, some ectopic neurogenic rosettes (circled) were observed. (B) Proliferating cells in E14.5 and E16.5 cortices were labeled with anti-Ki67 (proliferation marker) antibody. (C,D) The fluorescence intensity of Tuj1 was measured and the number of Ki67^+^ cells counted, followed by normalization to the area of analysis, and are shown as fold changes compared with the E14.5 control cortex. Note the increase in Tuj1^+^ cells and decrease in Ki67^+^ cells at E14.5 in the Arpc2-deficient cortex. Data shown represent mean±s.e.m. (*n*=5); *P<0.01, compared with control (ANOVA). CP, cortical plate; SVZ, subventricular zone; VZ, ventricular zone. Scale bars: 50 µm.

To examine the cellular organization of the Arpc2-deficient embryonic cortex, we performed immunostaining of nestin (neural progenitor marker) and Tuj1 (neuronal marker). In the control, as neurogenesis first begins nestin-positive RGCs are confined to the VZ as newly born neurons migrate to the outer layer of the cortex. By contrast, disorganized structures with ectopic neurogenic rosettes were present in the Arpc2-deficient cortex (Fig. 1A). In addition, there were significant numbers of Tuj1-positive neurons lining the ventricular surface. *Arpc2* deletion significantly increased the number of Tuj1-positive neurons at E14.5 (Fig. 1C). Interestingly, whereas E16.5 control cortex exhibited increased Tuj1-positive neurons compared with E14.5 (*P*<0.01), there was no further increase in Tuj1-positive neurons in the Arpc2-deficient cortex between E14.5 and E16.5.

To examine whether proliferating neural progenitors were depleted following *Arpc2* deletion, we immunostained for Ki67, which is a well-established marker for mitotic cells. *Arpc2* deletion significantly decreased the number of Ki67-positive cells at E14.5 (Fig. 1B,D). These results suggested premature neuronal differentiation and depletion of neural progenitors following *Arpc2* deletion. Since DAPI staining suggested widely occurring cell death in the Arpc2-deficient cortex, we next examined if Arpc2-deficient cortex exhibited increased apoptosis by cleaved caspase 3 immunoreactivity. Whereas apoptotic cells were not detected in the control cortex, there was a significant number of apoptotic cells in the Arpc2-deficient cortex (Fig. S3).

### The Arp2/3 complex is required for rapid extension and stability of RGC basal processes

**Fig. 2.**
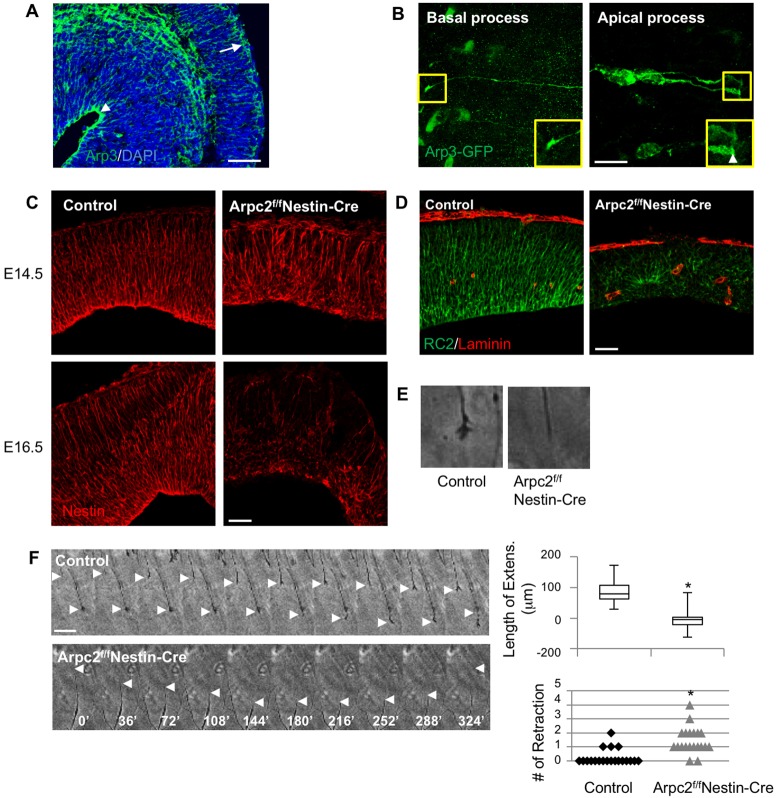
**Impaired RGC basal processes following *Arpc2* deletion.** (A) Immunohistochemical localization of endogenous Arp3 at E16.5 indicates Arp3 expression throughout the developing cerebral wall. Arp3 immunoreactivity was enriched at the apical and basal sides of RGCs. Arrowhead, apical end-feet; arrow, basal end-feet. (B) RGCs in the E14.5 control cortex were electroporated with Arp3-GFP DNA followed by immunostaining with anti-GFP antibody. Arp3 was expressed throughout the RGC basal and apical processes as well as cell soma. It was enriched at the basal end-feet (left panel) and the apical end-feet (right panel, arrowhead). (C) RGCs in E14.5 and E16.5 cortices were labeled with anti-nestin antibody. In the control cortex, polarized RGCs span the width of the cerebral wall. By contrast, in the Arpc2-deficient cortex the polarized organization of RGCs is severely disrupted. At E14.5, many RGC processes were not extended from the ventricular surface, but from the ectopic rosettes. At E16.5, the majority of the Arpc2-deficient RGC processes were short and misoriented. (D) E14.5 cortices were stained with RC2 (RGC marker) and anti-laminin (pia basement membranes) antibodies. The RGC processes in Arpc2-deficient cortex are short and disoriented (many extended from the rosette). (E,F) *Ex vivo* RGC process outgrowth analysis. Control and Arpc2-deficient cortices were embedded in 100% Matrigel and live imaged. (E) Arpc2-deficient RGC processes are pointed and the dynamic raffles are absent. (F) Montages depicting process outgrowth (arrowheads) of control and Arpc2-deficient RGCs (time in min). The net length of extension and the number of retractions in the control and Arpc2-deficient RGC processes within a 6 h time-lapse recording were measured. Box plot shows the net length of extension of control RGCs (*n*=20; median 78.8, range 29.8-173.4) and that of Arpc2-deficient RGCs (*n*=20; median –3.73, range –61.2-82.5). *P<0.01, compared with controls (ANOVA). Note that the shorter length of extension in Arpc2-deficient compared with control RGC processes is due to frequent retractions. Scale bars: 50 µm in A,C,D; 10 µm in B; 25 µm in F.

The above phenotypes are consistent with a role for the Arp2/3 complex in RGC morphogenesis. Next, we examined the pattern of localization of the Arp2/3 complex in RGCs throughout cortical neurogenesis (E12.5-16.5) by immunofluorescent staining for the Arp3 subunit. We found that Arp3 was enriched both at the apical and the basal sides of RGCs (Fig. 2A). We also introduced Arp3-GFP into RGCs in E14.5 cortex by *ex utero* electroporation to visualize the Arp2/3 complex in individual RGC processes. Localization of Arp3-GFP can be seen throughout the apical and basal processes as well as the cell soma and nucleus, but it was enriched at both basal and apical end-feet, especially at the ventricular surface (Fig. 2B). These observations suggest that the Arp2/3 complex might have multiple roles in RGCs.

To further characterize RGC defects resulting from *Arpc2* ablation, we used anti-nestin and RC2 antibodies as markers to assess the morphology of the RGC processes. At E14.5, the control developing cortex showed the typical radial organization of the RGC processes that span the width of the cerebral wall, whereas the Arpc2-deficient cortex exhibited disorganized processes (Fig. 2C). This defect was exacerbated at a later stage (E16.5), when the remaining RGCs extended short, misoriented processes and the entire RGC scaffold was drastically abnormal (Fig. 2C). Laminin immunostaining was performed to assess the anchorage of RGCs, labeled by RC2, to the basement membrane (Fig. 2D). Laminin-positive basement membrane, although somewhat discontinuous, was present in the Arpc2-deficient cortex, yet most of the RGC basal processes were not attached to the basement membrane. Therefore, these results suggest that the defective RGC process extension is not due to the lack of basement membrane but is more likely to be due to a lack of normal process extension per se.

To directly examine RGC process extension, control and Arpc2-deficient embryonic cortices were embedded in Matrigel and the *ex vivo* RGC process extension was recorded by time-lapse microscopy. Similar approaches have been used to study glia-guided neuronal migration (Edmondson and Hatten, 1987; Voss et al., 2008; Wichterle et al., 1999). Tip morphologies evidently differed: whereas wild-type RGC basal leading edges were dynamic and exhibited a growth cone-like morphology, most leading processes in mutant RGCs were pointed and lacked dynamic membrane ruffles (Fig. 2E, Movies 1,2). In addition, control RGCs extended their basal processes steadily, with an average speed of 0.24±0.1 µm/min (Fig. 2F, Movies 1,2). Arpc2-deficient RGCs extended slightly faster, with an average speed of 0.33±0.1 µm/min. However, the final average length of extension of Arpc2-deficient RGC processes was much shorter than that of control RGC processes over a 6-h duration, as a result of frequent retraction. These results suggest that the Arp2/3 complex is not only required for the formation of the growth cone-like structure at the tip of the basal process but is also crucial for the stability of the extended processes.

Surprisingly, in addition to an impairment in RGC basal process outgrowth, we also found that the lumen of blood vessels (laminin-positive endothelial cells) in the Arpc2-deficient cortex was enlarged (Fig. S4B) and the pia basement membrane (also labeled by laminin) was broken, suggesting blood vessel malformation during corticogenesis. To rule out the possibility that blood vessel malformation is due to Cre expression and *Arpc2* knockout in the endothelial cells (which also express nestin), we analyzed the ultrastructure of the blood vessels in *Arpc2^f/f^ Emx1-Cre* cortex. The lumen of the capillary was also enlarged and the capillary walls lined by endothelial cells in the *Arpc2^f/f^ Emx1-Cre* cortex were stretched and thin compared with those in the control cortex (Fig. S4C,D). This finding might explain the observed IVH phenotype, and is consistent with a recent report that impaired RGC functions lead to abnormal brain angiogenesis and neonatal cerebral hemorrhage (Ma et al., 2013).

### Loss of Arpc2 results in disrupted RGC polarity and adhesion

**Fig. 3.**
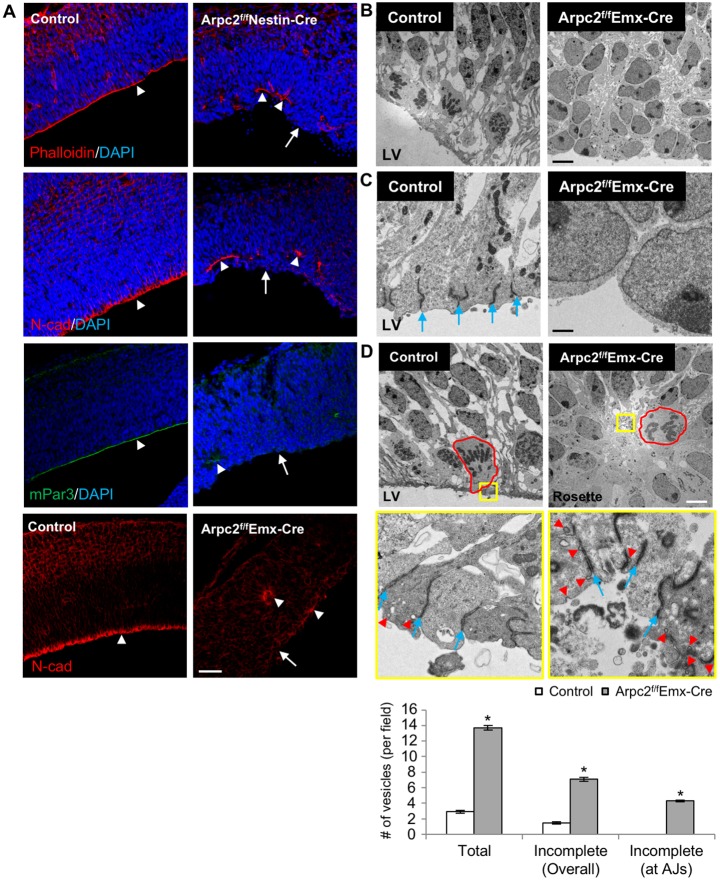
**Disrupted RGC apical adhesion following *Arpc2* deletion.** (A) E14.5 cortices were immunostained with anti-N-cadherin (AJ) or anti-Par3 (apical polarity marker) antibodies or stained with phalloidin (F-actin). Note the loss of actin network, AJs and apical polarity marker staining as a continuous line, but present ectopically, following *Arpc2* deletion. Arrowhead indicates AJ components; arrow indicates the apical surface. (B-D) TEM analysis of control and Arpc2-deficient VZ at E14.5. (B,C) Control VZ exhibited the typical columnar organization and AJs at the ventricular surface. (D) Arpc2-deficient VZ lacked the columnar organization and exhibited neurogenic rosettes. The center of rosettes contained mitotic cells (red outlines) and tangled AJs (see yellow boxed region, as magnified beneath). There is an accumulation of vesicles (red arrowheads) at the AJs (blue arrows) and apical surface. The size and number of the vesicles were determined. Values represent mean±s.e.m. from ten random fields. *P<0.01, compared with controls (ANOVA). There is an increase in total vesicle numbers as well as in incomplete vesicles at the apical end-feet in the Arpc2-deficient cortex. Also note the significant increase in incomplete vesicles at AJs in the Arpc2-deficient cortex. LV, left ventricle. Scale bars: 50 µm in A; 5 µm in B,D; 500 nm in C.

The enrichment of the Arp2/3 complex at the apical end-feet of RGCs indicates that it might play a role in the function of apical end-feet, especially given that the Arp2/3 complex has been shown to be involved in AJ formation and maintenance in cultured epithelial cells (Han et al., 2014; Tang and Brieher, 2012), as well as in the maintenance of PAR asymmetry in *C. elegans* early embryos (Shivas and Skop, 2012). AJ components such as N-cadherin and F-actin, as well as apical polarity proteins such as Par3 (Pard3), form a continuous apical band in the control cortex (Fig. 3A). By contrast, at E14.5 in the Arpc2-deficient cortex the apical surface was largely devoid of enrichment for these proteins, despite some abnormal accumulation. To further examine whether AJs and, consequently, RGC apical polarity were affected in the Arpc2-deficient cortex, we analyzed the ultrastructural organization of RGCs by thin-sectioning transmission electron microscopy at E14.5. A pseudostratified neuroepithelium was observed in the control cortex (Fig. 3B,D). While mitotic cell nuclei migrated to the ventricular surface, the nuclei of the elongated interphase cells were localized some distance away from the ventricular surface and these cells maintained connections with their neighbors by AJs through their apical end-feet. In the Arpc2-deficient cortex, most RGCs lacked AJs or polarized alignment (Fig. 3B,C). Interphase, but not mitotic, nuclei were frequently seen to localize at the apical surface. Disorganized AJs and mitotic cells were instead ectopically located in the rosettes in both VZ and subventricular zone (SVZ) (Fig. 3D).

The Arp2/3 complex nucleates actin filaments for endocytic vesicle scission, a process important for E-cadherin-mediated AJ formation. Indeed, more vesicles accumulated in the Arpc2-deficient cortex near the AJs or the apical membrane of RGC apical end-feet that were ectopically located within the center of rosettes (Fig. 3D). We quantified the number of vesicles at the apical end-feet and showed that endocytic vesicles were increased in the mutant RGCs compared with the control (Fig. 3D). Most of these vesicles remained attached to the plasma membrane, especially at the AJs, in the mutant but not in control RGCs, consistent with a failure in the scission step of endocytosis.

### Loss of Arpc2 results in altered cell fate and disorganized cortical layers

**Fig. 4.**
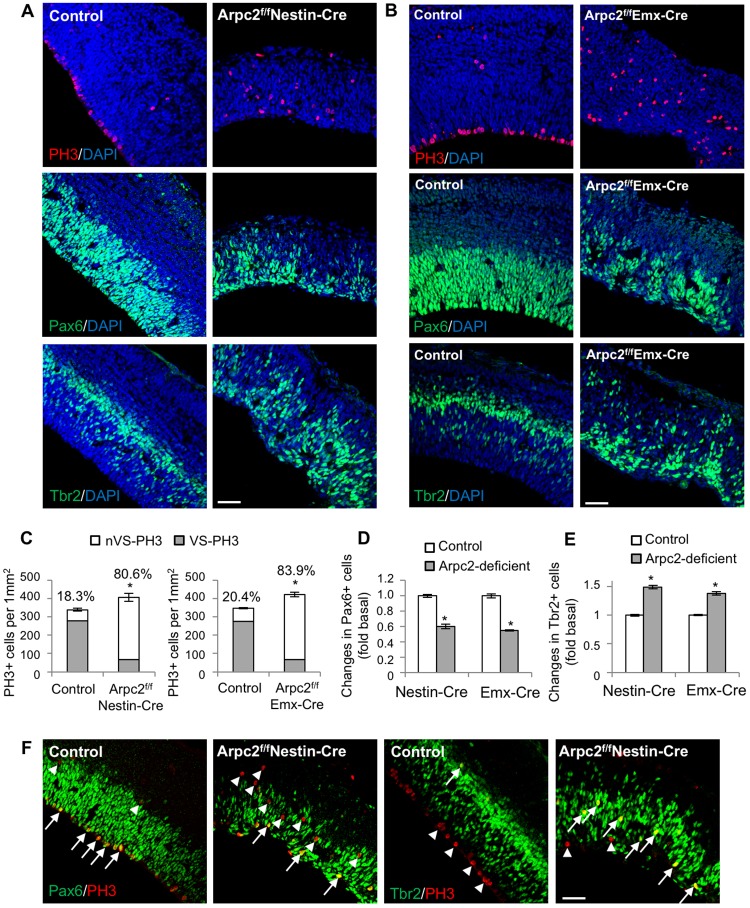
**Abnormal mitotic entry location and altered cell fate in Arpc2-deficient cortex.** (A,B) E14.5 cortices were immunostained with anti-phospho-histone H3 (PH3), anti-Pax6 (RGC marker) or anti-Tbr2 (IPC marker) antibodies. (C) The number of PH3^+^ cells lining the ventricular surface (VS-PH3) or localized at the basal position (nVS-PH3) were counted and normalized to the area of analysis. There is an increase in the percentage of nVS-PH3^+^ cells in the Arpc2-deficient cortex. (D,E) The number of Pax6^+^ cells and Tbr2^+^ cells were counted and normalized to the area of analysis, and are shown as fold change compared with control cortex. Note the decrease in Pax6^+^ and the increase in Tbr2^+^ cells in the Arpc2-deficient cortex. Data shown are mean±s.e.m. (*n*=4); *P<0.01, compared with controls (ANOVA). (F) E14.5 cortices were immunostained with anti-PH3, anti-Pax6 or anti-Tbr2 antibodies. Arrows, PH3 double-positive (with Pax6 or with Tbr2) cells; arrowheads, PH3 single-positive cells. There is an increase in Tbr2/PH3 double-positive cells in the Arpc2-deficient cortex. Scale bars: 50 µm.

Since the Arp2/3 complex is required for the maintenance of AJs, which have been shown to affect the fate of neural progenitor cells (reviewed by Kim and Walsh, 2007), we next analyzed whether the Arpc2-deficient RGCs are able to maintain their stem cell identity or adopt an IPC fate. As RGCs and IPCs divide at apical and basal positions, respectively, observation of both progenitor populations can be accomplished by immunolabeling for phospho-histone H3 (PH3). *Arpc2* deletion significantly reduced the number of PH3-positive cells lining the ventricular surface, and increased the number of PH3-positive cells that divide at more basal positions (Fig. 4A-C). Staining of Pax6, an RGC marker, or Tbr2 (Eomes), an IPC marker, showed that *Arpc2* deletion leads to a reduction in the number of RGCs (Fig. 4A,B,D) and a concomitant increase in the number of IPCs, the distribution of which extends all the way to the ventricular surface (Fig. 4A,B,E). In fact, most of the dividing cells in the basal area of the Arpc2-deficient cortex were Tbr2-positive IPCs (Fig. 4F). These results suggest that the Arp2/3 complex is not required for the division of RGCs or IPCs but is crucial for maintaining RGC identity. As expected, at E16.5 the control cortex showed decreased numbers of RGCs compared with those at E14.5. Interestingly, there were fewer RGCs in the Arpc2-deficient than in the control cortex at both time points (Fig. S5A,C). The number of IPCs in the Arpc2-deficient cortex increased at E14.5 but decreased at E16.5, as compared with the largely unchanged control (Fig. S5B,D). Based on the above findings, the reduction in the number of RGCs might be due to exhaustion of RGCs as a result of premature differentiation, decreased proliferation and/or increased apoptosis.

### Loss of Arpc2 results in disrupted cortical lamination and impaired neurogenesis

**Fig. 5.**
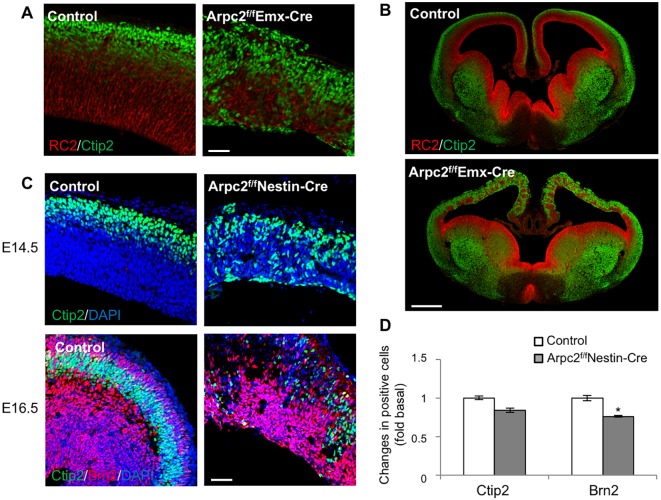
**Impaired neurogenesis and disrupted cortical lamination following *Arpc2* deletion.** (A,B) E14.5 cortices were labeled with RC2 (RGC marker) and anti-Ctip2 (layer V) antibodies. Note the disorganized RGC processes and the cortical neurons in the dorsal region of the *Arpc2^f/f^ Emx1-Cre* cortex, but not in the ventral region. (C) E14.5 and E16.5 cortices were immunostained with anti-Ctip2 (layer V) or anti-Brn2 (layer II-III) antibodies. Note the neurons lining the ventricular surface or throughout the cerebral wall in the mutant cortex, in contrast to the control cortex. (D) The fluorescence intensities of Ctip2 and Brn2 at E16.5 were quantified followed by normalization to the area of analysis, and are shown as fold change compared with control cortex. There is a decrease in Ctip2^+^ and Brn2^+^ cells at E16.5 in the Arpc2-deficient cortex. Data shown are mean±s.e.m. (*n*=5); *P<0.05, compared with controls (ANOVA). Scale bars: 50 µm in A,C; 500 µm in B.

Disruption in final neuronal positions was evident in the *Arpc2^f/f^ Emx1-Cre* cortex as early as E14.5, even though there was no apparent enlargement in the lateral ventricles in these mutants (Fig. 5A,B). To examine cortical lamination following *Arpc2* deletion, newly generated cortical neurons were stained for the cortical layer-specific markers Ctip2 (Bcl11b) and Brn2 (Pou3f2). In the control cortex, Ctip2^+^ and Brn2^+^ neurons migrated to distinct laminar positions (Fig. 5C). Both Ctip2^+^ and Brn2^+^ neurons were generated in the Arpc2-deficient cortex, but the laminar organization of the neurons in the Arpc2-deficient cortex was severely disrupted. At E16.5, the number of Brn2^+^ neurons was significantly reduced in the Arpc2-deficient cortex, but the decrease in the number of Ctip2^+^ neurons in the Arpc2-deficient cortex was not statistically significant (Fig. 5D).

### The Arp2/3 complex has a cell-autonomous role in neuronal migration

**Fig. 6.**
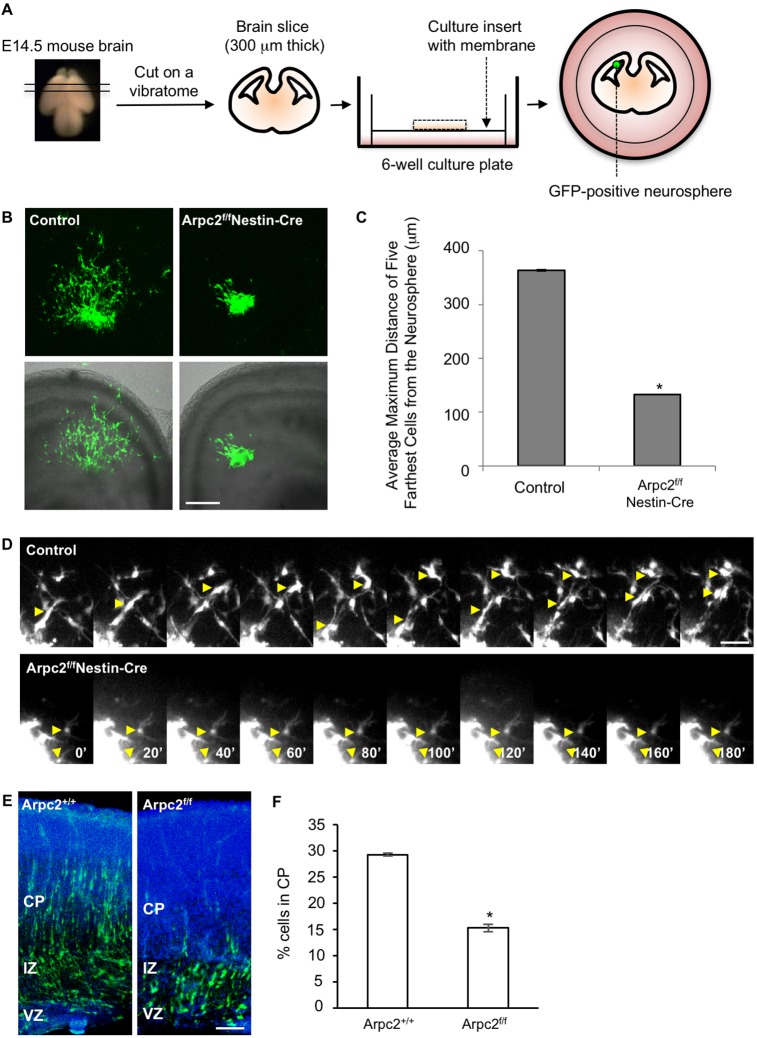
**Arpc2-deficient neuronal precursors fail to migrate in *ex vivo* brain slice culture.** (A) The organotypic brain slice co-culture system. E14.5 mouse brain was embedded in 4% agarose and slices (300 µm thick) prepared on a vibratome. The brain slices were then cultured on an insert in a 6-well plate. GFP was introduced into the control and Arpc2-deficient cortical neural progenitors by lentiviral transduction. GFP-positive cells were sorted and cultured as neurospheres and placed on the ventricular surface of the wild-type brain slices. (B) Shown are fluorescence images of migrating neuronal precursors (GFP positive) after 24 h of co-culture (top panels), and combined fluorescence/transmitted light micrographs of brain slice co-cultures (bottom panels). (C) Maximum distance of neuronal progenitor migration out of the neurospheres. Values represent mean±s.e.m. of the five longest migrations from eight transplanted neurospheres (*n*=40) from three independent experiments. (D) Montages showing the migration of two control neuronal precursors (yellow arrowheads), as well as two Arpc2-deficient neuronal precursors that failed to move. Time in min. (E) Representative E18.5 coronal sections after *in utero* electroporation at E14.5 with Dcx-Cre-iGFP plasmid. Electroporated cells were visualized with anti-EGFP (green) and nuclei were stained with DAPI (blue). (F) Values represent the mean±s.e.m. percentage of electroporated cells in the cortical plate (*n*=7). *P<0.01, compared with controls (ANOVA). IZ, intermediate zone. Scale bars: 200 µm in B; 25 µm in D; 100 µm in E.

The observed neuronal misplacement could be due to disruptions in RGC scaffolding or defects in neuronal motility. The Arp2/3 complex controls actin nucleation at the leading edge of migrating fibroblasts (reviewed by Bisi et al., 2013; Pollard, 2007; Suraneni et al., 2012; Wu et al., 2012), but the function of the Arp2/3 complex in neuronal migration has not been determined. However, the neuronal migration defect in *Arpc2* mutant cortex could simply result from short and disorganized RGC processes rather than reflecting a cell-autonomous role for the Arp2/3 complex in migrating neuronal precursors. To distinguish between these two possibilities, we used an *ex vivo* brain slice culture system with implementation of neurospheres cultured from control and mutant animals (Fig. 6A). The combination allowed us to examine how the Arpc2-deficient neuronal precursors migrate in the wild-type brain environment, which has normal and polarized RGCs. The control neurosphere-derived neuronal precursors were able to migrate towards the CP (Fig. 6B,C, Movies 3,4). Strikingly, the Arpc2-deficient neurosphere-derived neuronal precursors failed to migrate toward the CP. This was not due to a defect in de-adhesion from the sphere, as even cells that exited the sphere and were able to extend and retract processes were unable to migrate (Fig. 6D, Movies 5,6).

Finally, to inactivate Arpc2 in migrating neurons *in vivo*, we electroporated doublecortin (*Dcx*) promoter-driven Cre and EGFP (Dcx-Cre-iGFP) into postmitotic but premigratory neurons in E14.5 embryos (Franco et al., 2011), which were then analyzed at E18.5. Inactivation of Arpc2 in mutant embryos resulted in significantly reduced numbers of neurons migrating toward the CP compared with the control wild-type embryos (Fig. 6E,F). Taken together, these results suggest that the Arp2/3 complex is required for neuronal migration by affecting both the migratory cells and their radial migration tracks formed by the RGC processes.

### The Arp2/3 complex is crucial for neuronal cells to migrate on soft or less adhesive substrates

**Fig. 7.**
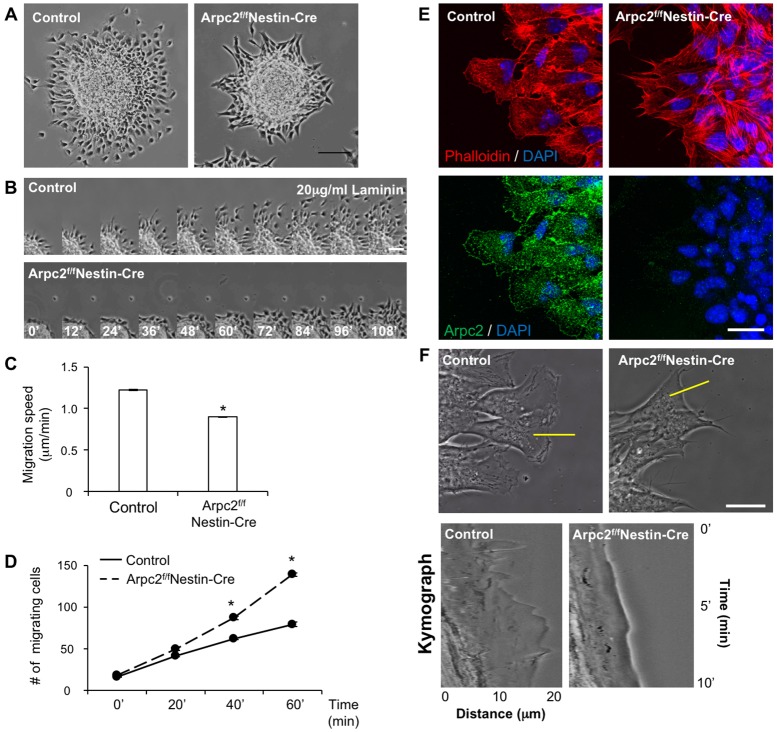
***In vitro* migration of Arpc2-deficient neural progenitors.** (A) *In vitro* neurosphere migration assay. Control and Arpc2-deficient neural progenitors migrated on 20 µg/ml laminin-coated glass-bottom dishes for 2 h. (B) Time-lapse montages of the migrating neural progenitors (time in min). (C) Mean±s.e.m. of the migration speed of six neural progenitors migrating out of each of six individual neurospheres (*n*=36). The Arpc2-deficient neural progenitors migrate more slowly than controls. (D) Mean±s.e.m. of the number of neural progenitors migrating out of six individual neurospheres (*n*=6). Neurospheres of similar size were used. (C,D) *P<0.01, compared with controls (ANOVA). (E) Localization of F-actin and endogenous Arpc2 in migrating neural progenitors. Arpc2-deficient neural progenitors are deficient in lamellipodia formation. Also note the localization of Arpc2 in lamellipodia in control but not in Arpc2-deficient neural progenitors. (F) High-magnification phase-contrast images of control and Arpc2-deficient neural progenitors (top row). Kymograph analysis along the yellow lines showing local protrusion-retraction cycles (bottom row). Note the less dynamic leading edge of the mutant cells compared with the control cells. Scale bars: 100 µm in A; 50 µm in B; 20 µm in E,F.

To further understand the mechanism by which loss of Arpc2 disrupts neural progenitor cell migration, we established a neurosphere migration assay with phase-contrast live imaging to monitor migration of the control and Arpc2-deficient neural progenitor cells on laminin-coated substrate (Fig. 7A). The Arpc2-deficient neural progenitors had the ability to migrate out of the sphere but they moved much more slowly than their control neural progenitor counterparts (Fig. 7B-D, Movies 7,8). Arpc2 immunostaining showed that the Arp2/3 complex localized in the lamellipodia of the migrating neural progenitors (Fig. 7E). Arpc2-deficient neural progenitors were unable to extend lamellipodia but they were able to generate filopodia-like protrusions. Consistently, the leading edge of Arpc2-deficient neural progenitors was less dynamic (Fig. 7F, Movies 9,10).

**Fig. 8.**
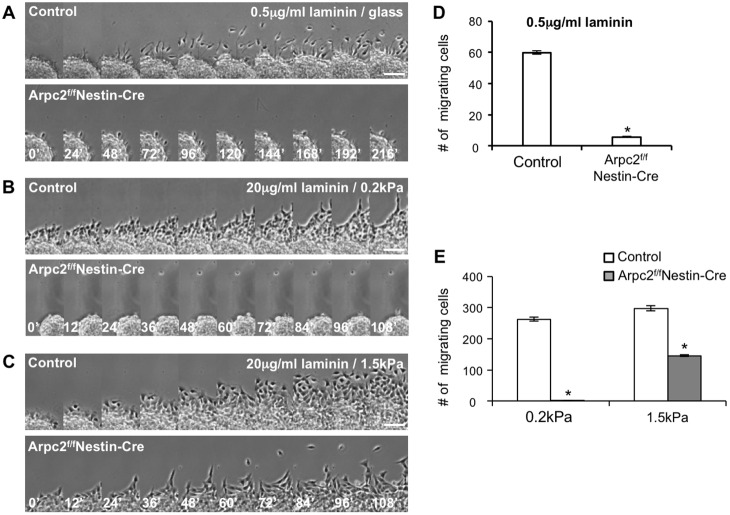
**Arpc2-deficient neural progenitors fail to migrate in response to low matrix adhesiveness and low matrix stiffness.** (A-C) Time-lapse montages of control and Arpc2-deficient neural progenitor migration on 0.5 µg/ml laminin-coated glass-bottom dishes (A) and 20 µg/ml laminin-coated elastic surface with a stiffness of 0.2 kPa (B) or 1.5 kPa (C). (D,E) Number of neural progenitors migrating out of the neurosphere during 6 h in A (D) or 2 h in B,C (E). Mean±s.e.m. (D, *n*=10; E, *n*=8). *P<0.01 versus controls (ANOVA). Arpc2-deficient neural progenitors failed to migrate under conditions of low matrix adhesiveness (0.5 µg/ml laminin) or on the soft (0.2 kPa) elastic surface. Scale bars: 50 µm.

Arpc2-deficient neural progenitors were motile and were able to migrate out of the neurospheres on the laminin-coated glass in this *in vitro* assay but not under the *ex vivo* condition. It is possible that the Arpc2-deficient neural progenitors were unable to respond properly to environmental cues. The physiological concentration of laminin is low in the embryonic brain (Liesi and Silver, 1988), and the brain is one of the softest tissues in the body (Moore et al., 2010; Spedden et al., 2012). Therefore, we examined whether the Arpc2-deficient neural progenitors would fail to migrate in the presence of low laminin concentrations (representing low matrix adhesiveness) and in conditions of reduced stiffness. Indeed, Arpc2-deficient neural progenitors lost their ability to migrate out of the spheres when they were plated on glass-bottom dishes coated with only 0.5 µg/ml, as opposed to 20 µg/ml, laminin (Fig. 8A,D, Movies 11,12). In addition, Arpc2-deficient neural progenitors lost their ability to migrate out of the spheres when they were plated on 20 µg/ml laminin-coated elastic surface with a low stiffness index of 0.2 kPa, whereas they retained the ability to migrate when the stiffness index was increased to 1.5 kPa (Fig. 8B,C,E, Movies 13-16). These results suggest a crucial role of the Arp2/3 complex in neuronal cell migration in the native brain environment, which is both soft and of low laminin concentration.

## DISCUSSION

Previous studies on the roles of the Arp2/3 complex in neural development have primarily focused on neuritogenesis, where it was shown that the Arp2/3 complex regulates axon growth cone actin dynamics and guidance, formation of axonal filopodia, and the development of dendritic spines (Korobova and Svitkina, 2008; Nakamura et al., 2011; Norris et al., 2009; Pinyol et al., 2007; San Miguel-Ruiz and Letourneau, 2014; Shakir et al., 2008; Spillane et al., 2011; Strasser et al., 2004; Wegner et al., 2008). Our study, using conditional *Arpc2* gene deletion in mice, has revealed multiple key roles for the Arp2/3 complex during mammalian corticogenesis. The Arp2/3 complex is required for efficient and stable extension of RGC basal processes, maintenance of RGC apicobasal polarity, which is likely to be through its role in AJ organization, and neuronal migration. Combined defects in these functions result in severely disrupted corticogenesis.

Emerging evidence has suggested that the RGC basal process has multiple roles in neurogenesis and neuronal migration (Kosodo and Huttner, 2009). Our data demonstrated that the Arp2/3 complex is required for a rapidly advancing growth cone-like leading edge of the RGC and, by inference, for the formation of a dendritic actin network, suggesting the motility mechanism might be similar to that of the neuronal growth cone (Korobova and Svitkina, 2008). It is interesting that the basal extension of Arpc2-deleted RGCs undergoes frequent retraction. This phenotype was also observed during neurite outgrowth of neurons isolated from the Arpc2-deficient cortex (P.-S.W. and R.L., unpublished observation). The Arp2/3 complex in some systems was shown to be recruited to microtubules and to play a role in microtubule dynamics (Oelkers et al., 2011; Saedler et al., 2004). Given that both the actin and microtubule networks and their interactions are crucial for neurite outgrowth (Gordon-Weeks, 2004), it is possible that Arp2/3 complex-mediated growth cone formation plays a role in stabilizing microtubules in RGC processes.

Another apparent effect of *Arpc2* deletion in RGCs is the loss of apical AJs and the PAR complex, leading to disruptions in neuroepithelial integrity and alterations in progenitor cell fate. This is consistent with the existing evidence that apical polarity and adhesion of RGCs are crucial for RGC self-renewal and the maintenance of the neurogenic niche. For example, it has been shown that deletion of *Cdc42*, a master regulator of cell polarity, results in retraction of apical processes and an abnormal IPC fate (Cappello et al., 2006). Other apical polarity proteins, such as Par3, regulate RGC asymmetric division and cell fate via Notch signaling (Bultje et al., 2009). Numb and Numbl inactivation leads to RGC dispersion and disorganized cortical lamination through regulating cadherin recycling (Rašin et al., 2007). Furthermore, N-cadherin-mediated AJs in RGCs regulate β-catenin signaling and cell fate (Zhang et al., 2010), and loss of cell-cell adhesion or apical polarity leads to a decrease in progenitor cell cycle re-entry (Chenn and Walsh, 2002) or an increase in proneural gene expression (Pierfelice et al., 2011), respectively. Conditional disruption (D6-Cre) of N-cadherin results in phenotypes similar to those of our *Arpc2* conditional mutant, such as disruption of the AJs localized in the apical end of RGCs, failure of RGCs to extend their bodies or processes between the VZ and the pia surface, as well as scattering of mitotic and postmitotic cells throughout the cortex (Kadowaki et al., 2007). Our observations are also in line with the finding in epithelial cells that WAVE2 (Wasf2) and the Arp2/3 complex are required for junctional integrity and tension (Han et al., 2014; Verma et al., 2012). In the epithelial cells, the Arp2/3 complex is regulated by cortactin and WAVE2, and disruption of these interactions or inhibition of Arp2/3 complex-mediated actin nucleation abolishes actin assembly at the AJs (Han et al., 2014; Tang and Brieher, 2012). Similar mechanisms could underlie the *in vivo* effect of *Arpc2* ablation on AJ organization at the ventricular surface.

Arp2/3 complex-mediated actin nucleation is also essential for endocytosis and exocytosis (Kaksonen et al., 2006; Toret and Drubin, 2006; Zuo et al., 2006). Vesicle trafficking has been shown to be crucial for cortical development and has also been linked to neurodevelopmental disorders (Sheen, 2012). The establishment and the maintenance of AJs require transport of cadherins from the trans-Golgi network to cell-cell contact regions, as well as the endocytic recycling of cadherins for continued dynamic remodeling of the AJs in response to tension and cell shape changes (Bryant and Stow, 2004; Lock and Stow, 2005; Paterson et al., 2003). Many of the vesicles that accumulated in the Arpc2-deleted RGCs were directly connected to the plasma membrane, consistent with a role for the Arp2/3 complex in endocytic vesicle scission. It is possible that the Arp2/3 complex maintains RGC apical polarity by regulating the endocytic recycling of AJ components. The Arp2/3 complex might also exert its effect on RGC fate by controlling apical trafficking of the Notch ligand Delta (Rajan et al., 2009), or by controlling the asymmetric distribution of the PAR complex (Shivas and Skop, 2012).

The Arp2/3 complex is required for the directional migration of fibroblasts during chemotaxis and haptotaxis (Suraneni et al., 2015, 2012; Wu et al., 2012) and for the directional migration of oligodendrocyte progenitor cells in response to the electric field (Li et al., 2015). However, the role of the Arp2/3 complex in migratory neurons and neural progenitors had not been investigated. Here, we demonstrated that Arpc2-deficient neurosphere-derived neuronal precursors and neural progenitors lose their ability to migrate in *ex vivo* brain slice culture. We also demonstrated that Arpc2-deficient migrating neurons fail to migrate to the CP *in vivo*. Our *in vitro* study suggests that Arpc2-deficient neuronal precursors and neural progenitors are intrinsically motile, similar to Arpc3-deficient fibroblasts and Arpc2-deficient oligodendrocyte progenitors. However, they fail to migrate in an environment of low matrix adhesiveness and stiffness, which is similar to the environment of the embryonic brain used for the *ex vivo* brain slice migration assay. This finding also implies that agents disrupting the Arp2/3 complex-based actin nucleation system might be particularly useful for impeding the precocious migratory ability of glioma cells in the tissue environment of the brain.

## MATERIALS AND METHODS

### Mice

Mice were cared for according to protocols approved by The Stowers Institute for Medical Research. Mice carrying an *Arpc2* allele flanked by loxP sites were generated by mating *Arpc2*^FRT and loxP^ mice with FLP mice (both obtained from the Wellcome Trust Sanger Institute, Hinxton, UK) to delete *lacZ* and *neo* between two FRT sites. All mouse strains used in this study were of the C57BL/6 background. *Arpc2^f/f^ Nestin-Cre* or *Arpc2^f/f^ Emx1-Cre* mouse embryos were generated by mating *Arpc2^f/f^* mice with either *Arpc2^lox/+^ Nestin-Cre*^+^ or *Arpc2^lox/+^ Emx1-Cre*^+^ mice. Littermate *Arpc2^f/f^ Nestin-Cre^−^* and *Arpc2^lox/+^ Nestin-Cre*^+^ mice are phenotypically normal and served as controls, as did *Arpc2^+/+^ Nestin-Cre*^+^ embryos. *Emx1-Cre* mice were obtained from The Jackson Laboratory.

### Immunohistochemistry, immunofluorescence and immunoblot analysis

Mouse embryonic brains were removed and fixed with 4% paraformaldehyde (PFA) in PBS overnight at 4°C followed by immunohistochemistry analysis. Neurospheres were plated on poly-D-ornithine and 20 µg/ml laminin-coated coverslips, allowed to migrate for 1 h and then fixed with 4% PFA in PBS for 15 min at room temperature followed by immunofluorescence analysis. Extracts from E13.5 and E15.5 cerebral cortices were prepared in RIPA lysis buffer and were subjected to immunoblot analysis. For details, including antibodies, see the supplementary Materials and Methods.

### *Ex utero* electroporation and preparation of brain slices

Approximately 2 µl DNA (2 µg/µl) were injected into the lateral ventricle and electroporated (five 50-ms pulses of 30 V at 950-ms intervals). Arp3-GFP plasmid (plasmid 8462: pEGFP-N1-ACTR3) was obtained from Addgene. Following electroporation, cortices were dissected, coronally sectioned (300 µm) in a vibratome (Leica), mounted on Millicell cell culture inserts (Millipore), and cultured in MEM/10% FBS (Invitrogen) for 1 h followed by Neurobasal medium with 2% B27 (Invitrogen) for 1 day.

### RGC process outgrowth assay

E14.5 mouse embryonic cortices were dissected and embedded in 100% Matrigel (BD Biosciences) and cultured in Neurobasal medium with 2% B27 for 1-2 days. RGC processes were then imaged by phase-contrast microscopy using a Nikon ECLIPSE TE2000-E inverted microscope attached to a live cell incubation chamber. Time-lapse images were processed with ImageJ (NIH).

### Transmission electron microscopy (TEM)

Mouse brain tissues were harvested and immersion-fixed in 2.5% glutaraldehyde for 2 h at room temperature. The tissues were washed in PBS and then processed for TEM. For details, see the supplementary Materials and Methods.

### Preparation of GFP-positive neurospheres

Neural progenitors were isolated from E14.5 embryonic cortices and cultured as neurospheres. Details of the procedure are provided in the supplementary Materials and Methods.

### *In vitro* neurosphere migration assay

Neurospheres were plated on 0.5-20 µg/ml laminin-coated glass-bottom dishes (MatTek Corporation), 20 µg/ml laminin-coated elastic surface with a stiffness of 0.2 kPa (Softview 35 mm/10 mm glass bottom, easy coat, Matrigen) or 20 µg/ml laminin-coated elastic surface with a stiffness of 1.5, 15 or 28 kPa (µ-Dish 35 mm, high, ESS Variety Pack, Ibidi). Neurospheres were allowed to attach to the bottom and then imaged during migration by phase-contrast microscopy as described above. Time-lapse images and kymographs were processed using ImageJ. Neurospheres of similar size (100-150 µm) were selected for quantification. Migration length was measured by selecting the center of mass throughout the length of the movie using the Chemotaxis and Migration tool for ImageJ (Ibidi). Migration length was then divided by time to obtain migration speed (µm/min). The number of migrating cells was counted using the ImageJ Cell Counter plugin. Migrating cells are defined as cells that protrude from the boundary of the neurosphere at indicated times.

### *In utero* electroporation

All surgeries were approved by the Institutional Animal Care and Use Committee (IACUC) and followed NIH guidelines for the ethical treatment of animals. *In utero* electroporation was performed as previously described (Rannals et al., 2016). In brief, timed pregnant dams (E14.5) were anesthetized by intraperitoneal injection of ketamine (100 mg/kg) and xylazine (10 mg/kg). A laparotomy was performed and the embryos were exposed. Plasmid DNA (pCIG2-DCX-CRE-IRES-GFP) was diluted to 1.5 µg/µl in PBS plus 1% Fast Green dye to visualize the injection. The plasmid mixture was then backfilled into a glass micropipette and DNA was injected into the left ventricle using a Picospritzer III pressure injector. A brief train of electrical pulses (BTX ECM8300, 35 V) was delivered to target dorsal cortices for transfection. Following surgery and after recovery from anesthesia, pregnant dams were housed in a vivarium (SoBran BioScience) and maintained on a 12 h light cycle and fed *ad libitum*. Four days after surgery, embryonic brains were harvested and immersed in 4% PFA overnight. Fixed brains were embedded in 2% low-melting agarose and 50 µm thick coronal sections were prepared using a vibrating microtome (Microm, HM 650V).
